# Biological Therapy in Inflammatory Bowel Disease Patients Partly Restores Intestinal Innate Lymphoid Cell Subtype Equilibrium

**DOI:** 10.3389/fimmu.2020.01847

**Published:** 2020-08-27

**Authors:** Brecht Creyns, Inge Jacobs, Bram Verstockt, Jonathan Cremer, Vera Ballet, Roselien Vandecasteele, Tim Vanuytsel, Marc Ferrante, Séverine Vermeire, Gert Van Assche, Jan L. Ceuppens, Christine Breynaert

**Affiliations:** ^1^KU Leuven Department of Microbiology, Immunology and Transplantation, Allergy and Clinical Immunology Research Group, Leuven, Belgium; ^2^KU Leuven Department of Chronic Diseases, Metabolism and Ageing, Translational Research Center for Gastrointestinal Disorders (TARGID), Leuven, Belgium; ^3^Department of Gastroenterology and Hepatology, KU Leuven, University Hospitals Leuven, Leuven, Belgium; ^4^Department of General Internal Medicine, KU Leuven, University Hospitals Leuven, Leuven, Belgium

**Keywords:** inflammatory bowel disease, innate lymphoid cells, circulation, biological therapy, CD, UC

## Abstract

Patients with Crohn disease (CD) and ulcerative colitis (UC) suffer from chronic relapsing intestinal inflammation. While many studies focused on adaptive immunity, less is known about the role of innate immune cells in these diseases. Innate lymphoid cells (ILCs) are recently identified cells with a high cytokine-producing capacity at mucosal barriers. The aim was to study the impact of biological treatment on ILC in CD and UC. Patients initiating anti–tumor necrosis factor (TNF), ustekinumab, or vedolizumab treatment were prospectively followed up and peripheral and intestinal ILCs were determined. In the inflamed gut tissue of patients with inflammatory bowel disease, we found an increase of ILC1 and in immature NKp44^−^ ILC3, whereas there was a decrease of mature NKp44^+^ ILC3 when compared to healthy controls (HCs). Similar but less pronounced changes in ILC1 were observed in blood, whereas circulating NKp44^−^ ILC3 were decreased. Fifteen percent of CD patients had NKp44^+^ ILC3 in blood and these cells were not detected in blood of HCs or UC patients. Therapy with three different biologicals (ustekinumab targeting the IL-12/23 cytokines, anti-TNF and vedolizumab) partly restored intestinal ILC subset equilibrium with a decrease of ILC1 (except for ustekinumab) and an increase of NKp44^+^ ILC3. Anti-TNF also mobilized more NKp44^+^ ILC3 in circulation. As ILC1 are proinflammatory cells and as NKp44^+^ ILC3 contribute to homeostasis of intestinal mucosa, the observed effects of biologicals on ILCs might contribute to their clinical efficacy.

## Introduction

Patients with inflammatory bowel diseases (IBDs), ulcerative colitis (UC) and Crohn disease (CD), suffer from an uncontrolled intestinal inflammation caused by interactions between genetic factors, the gut microbiome, environmental factors and the immune system (1, 2). In the last decades, a lot of attention has been focused on the involvement of the adaptive immune system, identifying CD as a T helper cell 1 (T_H_1) and T_H_17 disease and UC as a T_H_2/T_H_17 disease (3–5). However, less is known about the involvement of innate immune cells in IBD pathogenesis. Innate lymphoid cells (ILCs) were identified as innate immune cells lacking antigen-specific receptors but having a high cytokine-producing capacity at mucosal barriers (6–8). Several subpopulations of ILCs were identified, sharing characteristics in their cytokine secretion pattern with the known T helper cell subpopulations. ILC1 share a typical transcription factor (T-bet) with T_H_1 cells and can produce interferon γ (IFN-γ); ILC2, similar to T_H_2 cells, are dependent on GATA-binding protein 3 (GATA-3) and produce type 2 cytokines interleukin (IL-4), IL-5, IL-9 and IL-13, whereas ILC3 are RAR-related orphan receptor γ-positive and produce IL-17 and IL-22 upon stimulation (9–11). These ILC3 can further be subdivided into natural cytotoxic receptor (NCR or NKp44)–negative and NCR-positive (NCR^+^) ILC3, which are enriched at the intestinal lining and produce IL-22 which are highly important in mucosal homeostasis (12). Nevertheless, this subdivision is not absolute as plasticity from ILC1 to ILC3 and vice versa and plasticity form ILC2 toward ILC1 has been observed *in vitro* (13, 14). NCR^−^ ILC3 have been identified as precursor ILCs with the capacity to differentiate into ILC1, ILC2 and NCR^+^ ILC3, depending on the stimulation conditions (15, 16).

In patients with active CD, an imbalance in intestinal ILCs has recently been observed, with a decrease of homeostatic ILC3 and an increase of proinflammatory ILC1 (17–19). As IL-12 and IL-23 might play distinct roles in this imbalance by stimulating ILC1 and ILC3 differentiation, respectively, we studied the effect of ustekinumab (which neutralizes the common p40 chain of IL-12 and IL-23) on ILC populations in blood and intestinal biopsies of IBD patients (20–22).

For comparison, we included patients on biological therapy with anti–tumor necrosis factor (TNF) and with vedolizumab in our study. These biologicals indeed are in theory less likely to interfere with the ILC subset balance.

As a second aim, we studied whether changes in ILC subsets in the circulation may reflect intestinal inflammation and whether differences could be observed between UC and CD patients. We analyzed if there is an effect of biological therapy on circulating ILCs and whether these correlate with the observed intestinal changes. Furthermore, we studied whether ILC levels in the circulation before initiation of biological therapy can predict response to treatment.

## Materials and Methods

### Patient Samples

Blood and tissue samples were collected using a prospective study protocol approved by the UZ Leuven Ethical Committee Review Board (S53684). Informed consent was obtained from healthy controls (who were included upon negative endoscopic findings during polyp screening) and from adult IBD patients with confirmed diagnosis of IBD. Consecutive IBD patients initiating biological therapy because of active endoscopic disease, were included and prospectively followed up with serial clinical assessment, C-reactive protein (CRP) and fecal calprotectin (fCAL) measurements (23, 24). All patients received drugs with standard dosage, according to the label.

Blood samples were collected at baseline prior to drug administration, as well as at weeks 0, 4, 8 and 14/24, by venipuncture in lithium heparin Vacutainers (BD, cat. #366480) and kept at room temperature until processing within 2 h. Tissue samples were collected during baseline endoscopic examination using biopsy forceps. At baseline, six mucosal colonic biopsies were taken at the edge of an inflamed ulcer, with follow-up biopsies taken at the same site. In healthy individuals, colonic biopsies were taken randomly in the sigmoid. Biopsies were collected in 10 mL sterile Roswell Park Memorial Institute 1640 medium (RPMI; Lonza) and stored at 4°C until processing (within 1 h). All endoscopies were performed by the same three IBD staff members (MF, SV, GVA).

### Outcome

Following national reimbursement criteria for biological therapies in UC, UC patients were endoscopically reassessed 8 (adalimumab) to 14 weeks (infliximab, vedolizumab) after treatment initiation. Likewise, all CD patients were endoscopically reevaluated 6 months after treatment initiation. In UC, endoscopic remission was defined as a Mayo endoscopic subscore of 0, whereas endoscopic improvement was defined as a Mayo endoscopic subscore ≤ 1. In CD, endoscopic remission was defined as a complete absence of ulcerations, whereas endoscopic improvement was considered in case of clear improvement (as compared to baseline) but with remaining ulcerative lesions (25).

### Blood Leukocyte Isolation

Peripheral blood mononuclear cells (PBMCs) were isolated with 50 mL Leucosep™ tubes (Greiner, VWR) according to manufacturer's instructions. In short, blood was diluted one to two with sterile phosphate-buffered saline (PBS) and centrifuged on top of 10 mL Lymphoprep (Stemcell Technologies) for 15 min at 1,200 relative centrifugal force. The leukocyte interphase was collected and washed twice in 50 mL of PBS with bovine serum albumin (BSA; ThermoFisher). Cells counts were determined using an ABX counter (Horiba, Japan). For cryopreservation, 10 × 10^6^ PBMCs were suspended in 10% dimethyl sulfoxide in cold fetal bovine serum (FBS) and placed in cryo containers at −80°C before transfer after 24h in liquid nitrogen. As cryopreservation and thawing of PBMCs resulted in a preferential loss of ILC3 (39.1 vs. 33.7% of total ILCs, *p* = 0.006) due to loss of CD117 expression also seen in CD117^+^ ILC2s, all samples were freshly isolated and stained (Supplementary Figure 1).

### Biopsy Leukocyte Isolation

Combined biopsies were washed in RPMI supplemented with 4-(2-hydroxyethyl)-1-piperazineethanesulfonic acid (HEPES; Lonza), 10% FBS (ThermoFisher) and 2% antibiotic-antimycotic (ThermoFisher). The epithelial layer was removed with RPMI supplemented with ethylenediaminetetraacetic acid (EDTA; Lonza), dithiothreitol (Chem-Lab, Belgium) and HEPES (Lonza) at 37°C on a magnetic stirrer at 400 revolutions/min (rpm) for 20 min. Biopsies were transferred to 5 mL of Hanks balanced salt solution with calcium and magnesium (HBSS^Ca2+/Mg2+^; Lonza) supplemented with HEPES, FBS, 5.0 mg collagenase IV (Worthington Biochemical, cat. LS004188, USA) and 10 μL DNase I (Roche, Germany) at 37°C on a magnetic stirrer at 400 rpm for 40 min. The cell suspension was strained through a 70-μm filter (EASYstrainer™; Greiner), quenched with 5 mL of PBS with 2.5% BSA, centrifuged at 400*g* for 5 min. The remaining undigested tissue was again resuspended in 5 mL of HBSS with Ca and Mg supplemented with HEPES, FBS, 5.0 mg collagenase and 10 μL DNase I at 37°C on a magnetic stirrer at 400 rpm for 20 min. Combined cell suspensions were counted using an ABX counter.

### Labeling of α4β7 Antibody

Human integrin α4β7/LPAM-1 (research-grade vedolizumab biosimilar) antibody (MAB10078-100, Novus Biologicals, UK) was coupled to Qdot 800 (ThermoFisher, S10455) according to manufacturer's instructions.

### Leukocyte Staining

Viability of isolated leukocytes was determined by incubation with Fixable Viability Dye 780 (FVD780, 65-0865-18; ThermoFisher) for 30 min in the dark before washing with PBS BSA. To identify ILCs and subsets, intestinal leukocytes and 4 × 106 PBMCs were stained with anti-CD11c (11-0116-42), anti-CD123 (11-1239-42), anti-CD14 (11-0149-42), anti-CD19 (11-0199-42), anti-CD1a (11-0019-42), anti-CD3 (11-0038-42), anti-CD303a (11-9818-42), anti-CD94 (11-0949-42), anti-FcεRIa (11-5899-42), anti-T cell receptor (TCR) αβ (11-9986-42), anti-TCR ɤδ (11-9959-42) and anti-CD34 (11-0349-42) (all fluorescein isothiocyanate), anti-CD45 Alexa Fluor 700 (AF700, 56-9459-42), anti-CD161 PE-Cy7 (25-1619-42), anti-CD4 eVolve 605 (83-0047; all ThermoFisher), anti-CD117 brilliant violet (BV)421 (562434; BD), anti-CD127 BV711 (351328), anti-CD294 AF647 (350104) and anti-CD336 PE (325108; all Biolegend). Stainings were performed in a total volume of 100 μL of PBS BSA supplemented with human serum and Brilliant Stain Buffer (563794; BD). To determine α4β7, β7 and CD69 expression, selected samples were additionally stained with anti-α4β7 Qdot-800, anti-β7 PE Dazzle 594 (104738; Biolegend) and anti-CD69 BV785 (310932; Biolegend). For intracellular cytokine detection, cells were first stimulated with Cell Activation Cocktail (423304; Biolegend) for 6 h. Intracellular staining of IL-4 (564113; BD), IL-17A (512326), IL-22 (366706) and IFN-γ (502526; all Biolegend) was performed according to manufacturer's instructions of the Foxp3 staining kit (00-5523-00; ThermoFisher). After staining, all cells were washed with PBS/BSA and fixated for 10 min in 0.5% paraformaldehyde. Cells were washed, resuspended in PBS/BSA/EDTA and stored at 4°C in the dark until acquisition.

### Flow Cytometry

Flow cytometry was conducted on a BD LSR Fortessa instrument in accordance with standard methods. Calibration was performed before each acquisition by CS&T beads (BD). At least 2 × 10^6^ events were acquired for PBMC samples. For analysis of leukocytes from biopsies, all events were acquired. For fluorescence compensation settings, single-color UltraComp eBeads™ compensation beads were used (ThermoFisher). Fluorescence Minus One (FMO) controls were included.

### Calprotectin and C-Reactive Protein Measurement

Fecal samples were collected at home and stored at 4°C until deposition within 24 h at the hospital; fCAL measurements were performed with the fCAL enzyme-linked immunosorbent assay kit (Bühlmann, Switzerland). C-reactive protein was determined in the blood by the clinical laboratory of the University Hospitals Leuven.

### Data Analysis and Statistics

FlowJo was used for cleaning up files, concatenating files and calculating manual gates and statistics. Doublets were carefully gated out in all samples. GraphPad Prism 8 (La Jolla, CA, USA) was used for statistical analysis; Shapiro–Wilk testing was performed for testing of normality. Paired analysis was performed by Wilcoxon matched-pairs signed rank testing, unpaired analysis by Mann–Whitney *U* testing and nonparametric Spearman correlation; ^*^p < 0.05, ^**^p ≤ 0.01 and ^***^p ≤ 0.001 were considered as significant.

## Results

### Patient Characteristics

Seventy-one IBD patients with active disease, 85 consecutive IBD patients initiating ustekinumab therapy, 25 patients initiating anti-TNF therapy and 32 patients initiating vedolizumab therapy were prospectively recruited. Demographic and clinical characteristics at baseline and endpoint are summarized in Tables 1–4. Patients initiating ustekinumab were all CD patients, whereas patients with active disease or initiating anti-TNF or vedolizumab treatment were a combination of UC and CD patients. The majority of patients initiating ustekinumab were refractory patients as evidenced by a longer disease duration (compared to the other treatment groups with anti-TNF and vedolizumab; 14.53 vs. 3.70 and 8.78 years, *p* < 0.001 and *p* = 0.005 respectively) and by previous exposure to anti-TNF or vedolizumab therapy (3.00 vs. 0.00 and 1.00 previous biologicals, both *p* < 0.001; Supplementary Figure 2).

**Table 1:** Demographic characteristics of patients with active disease and healthy controls.

	**PBMC**		**Biopsies**	
	**IBD**	**HCs**	**IBD**	**HCs**
No. of patients, *n*	45	22	26	17
UC/CD, *n* (%)	21/24 (44.00%)	0 (0.00%)	11/15 (42.30%)	0 (0.00%)
Sex, women, *n* (%)	25 (54.00%)	12 (63.60%)	17 (65.00%)	10 (58.80%)
Age, median (IQR), year	34 (25.7; 52.0)	26 (23.5; 38.5)	37 (26.5; 49.0)	59 (49.0; 64.0)
Disease duration, median, year (IQR)	10.80 (4.85; 12.52)		10.8 (3.37; 13.74)	
C-reactive protein baseline, median (IQR), mg/mL	6.10 (0.45; 15.00)		4.40 (1.60; 16.00)	
Fecal calprotectin, median (IQR), μg/g	1,478 (138; 1,800)		1,324 (77; 1,800)	
SES-CD, median (IQR)	3.5 (0.8; 10.0)		2.5 (2.0; 3.0)	
Mayo score UC baseline, median (IQR)	2 (2;2)		2 (2;3)	
**Disease location**, ***n*** **(%)**				
Ileal (L1)	4 (16.00%)		4 (26.70%)	
Colonic (L2)	3 (12.50%)		3 (20.00%)	
Ileocolonic (L3)	17 (70.80%)		8 (53.30%)	
Upper GI involvement (L4)	0		0	
**Disease extension**, ***n*** **(%)**				
Proctitis (E1)	3 (14.30%)		1 (9.00%)	
Left-sided (E2)	4 (19.00%)		2 (18.20%)	
Pancolitis (E3)	14 (66.70%)		8 (72.70%)	

*Mann–Whitney U testing between IBD patients and HCs; PBMC, peripheral blood mononuclear cell; IBD, inflammatory bowel disease; HCs, healthy controls; UC, ulcerative colitis; CD, Crohn disease; CRP, C-reactive protein; fCAL, fecal calprotectin; SES-CD, simple endoscopic score CD; GI, gastrointestinal; IQR, interquartile range*.

**Table 2:** Characteristics of patients initiating ustekinumab treatment at baseline (week 0) and endpoint (week 24).

	**Ustekinumab**			
	**PBMC**		**Biopsies**	
	**Responders**	**Nonresponders**	**Responders**	**Nonresponders**
No. of patients, *n*	15	70	10	37
UC/CD, *n* (%)	0/15 (0%)	0/70 (0%)	0/10 (0%)	0/37 (0%)
Sex, women, *n* (%)	12 (80.00%)	45 (64.29%)	7 (70.00%)	22 (59.46%)
Age, median (IQR), year	30.20 (24.50; 44.95)	43.8832.86; 55.54)*	31.37 (7.28; 43.93)	41.57 (32.84; 51.02)
Disease duration, year, median (IQR)	8.45 (3.39; 22.80)	15.2 (9.44; 24.75)	10.37 (5.35; 23.16)	15.33 (10.34; 25.94)
Previous biologicals, median (IQR)	2 (1;3)	3 (2;3)**	2 (1;3)	3 (2;3)
Steroids at start, *n* (%)	6 (40.00%)	15 (21.43%)	5 (50.00%)	10 (27.03%)
CRP baseline, median (IQR), mg/mL	16.4 (4.5; 24.8)	7.0 (3.6; 16.9)	26.6 (7.3; 56.0)	6.6 (2.7; 14.0)
CRP at endoscopic evaluation, median (IQR), mg/mL	5.9 (1.67; 9.15)	7.0 (3.6; 16.9)	1.6 (0.6; 8.2)	6.3 (3.4; 18.4)
fCAL baseline, median (IQR), μg/g	1,800 (445; 1,800)	678 (84; 1,800)	1,800 (169; 1,800)	1,800 (680; 1,800)
fCAL at endoscopic evaluation, median (IQR) μg/g	171 (36; 1,119)	1,800 (492; 1,800)*	472 (106; 1,119)	1,800 (492; 1,800)
**Disease location**, ***n*** **(%)**				
Ileal (L1)	4 (26.67%)	21 (30.00%)	2 (20.00%)	10 (27.03%)
Colonic (L2)	2 (0.133%)	8 (0.11%)	2 (20.00%)	6 (16.22%)
Ileocolonic (L3)	9 (0.60%)	41 (58.57%)	6 (60.00%)	21 (56.76%)
Upper GI (L4)	0 (0.00%)	0 (0.00%)	0 (0.00%)	0 (0.00%)
**Disease behavior**, ***n*** **(%)**				
B1	10 (66.67%)	19 (27.14%)	6 (60.00%)	11 (29.73%)
B2	3 (20.00%)	30 (42.86%)	3 (30.00%)	17 (45.95%)
B3	2 (0.13%)	19 (27.14%)	1 (10.00%)	9 (24.32%)

Response was defined as a minimal 50% of SES-CD. Mann–Whitney U testing between responders and responders; *p < 0.05, **p ≤ 0.01. PBMC, peripheral blood mononuclear cell; CD, Crohn disease; CRP, C-reactive protein; fCAL, fecal calprotectin; SES-CD, Simple Endoscopic Score CD; GI, gastrointestinal; IQR, interquartile range.

**Table 3:** Demographic characteristics of patients on anti-TNF treatment.

	**Anti-TNF**			
	**PBMC**		**Biopsies**	
	**Responders**	**Nonresponders**	**Responders**	**Nonresponders**
No. of patients (*n*)	13 (52.00%)	12 (48.00%)	5 (33.33%)	10 (66.66%)
UC/CD, *n* (%)	3/10 (23.08%)	7/5 (58.33%)	1/4 (20.00%)	6/4 (60.00%)
Sex, women, *n* (%)	7 (53.85%)	7 (58.33%)	2 (40.00%)	8 (80.00%)
Age, median (IQR), year	33.0 (27.0; 46.5)	28.5 (21.3; 47.3)	47.0 (33.0; 68.5)	23.0 (20.8; 40.8)*
Disease duration, median (IQR), year	3.7 (1.3; 8.9)	3.7 (1.3; 5.9)	4.1 (1.9; 4.1)	3.45 (1.2; 4.9)
Previous biologicals, median (IQR)	0 (0; 0)	0 (0; 0)	0 (0; 1)	0 (0; 1)
Steroids at start, *n* (%)	7 (53.85%)	7 (58.33%)	3 (60.00%)	7 (70.00%)
C-reactive protein baseline, median (IQR), mg/ml	4.8 (0.9; 52.7)	19.1 (5.3; 42.5)	66.0 (0.9; 80.9)	7.4 (2.3; 33.8)
C-reactive protein endpoint, median (IQR), mg/ml	0.7 (0.3; 2.9)	4.5 (0.7; 15.6)*	1.4 (0.475; 3.0)	7.2 (0.9; 17.8)
Fecal calprotectin baseline, median (IQR), μg/g	225 (0; 1,800)	1,800 (670; 1,800)	170 (7; 1,800)	1,800 (1,149; 1,800)
Fecal calprotectin endpoint, median (IQR), μg/g	0 (0; 76)	575 (0; 1,617)*	23 (0; 243)	1,069 (669; 1,800)*
**Disease location**, ***n*** **(%)**				
Ileal (L1)	1 (10.00%)	0 (0.00%)	2 (50.00%)	3 (75.00%)
Colonic (L2)	5 (50.00%)	3 (60.00%)	2 (50.00%)	1 (25.00%)
Ileocolonic (L3)	4 (40.00%)	2 (40.00%)	0 (0.00%)	0 (0.00%)
Upper GI involvement (L4)	0 (0.00%)	0 (0.00%)	0 (0.00%)	0 (0.00%)
**Disease extension**, ***n*** **(%)**				
Proctitis (E1)	1 (33.33%)	3 (42.86%)	0 (0.00%)	4 (66.67%)
Left-sided (E2)	2 (66.60%)	1 (14.29%)	1 (100.00%)	0 (0.00%)
Pancolitis (E3)	0 (0.00%)	3 (42.86%)	0 (0.00%)	2 (33.33%)
**Biological**				
Infliximab	6 (46.15%)	3 (25.00%)	2 (40.00%)	2 (20.00%)
Adalimumab	5 (38.46%)	8 (66.67%)	2 (40.00%)	6 (60.00%)
Golimumab	2 (15.38%)	1 (8.33%)	1 (20.00%)	2 (20.00%)

Mann–Whitney U testing between responders and nonresponders; *p < 0.05, considered as significant. PBMC, peripheral blood mononuclear cell; UC, ulcerative colitis; CD, Crohn disease; GI, gastrointestinal; IQR, interquartile range.

**Table 4:** Demographic characteristics of patients on vedolizumab treatment.

	**Vedolizumab**			
	**PBMC**		**Biopsies**	
	**Responders**	**Nonresponders**	**Responders**	**Nonresponders**
No. of patients (*n*)	14 (43.75%)	18 (56.25%)	11 (52.48%)	10 (47.62%)
UC/CD, *n* (%)	8/6 (57.14%)	5/13 (27.78%)	7/4 (63.63%)	4/6 (40.00%)
Sex, women, *n* (%)	8 (57.14%)	11 (61.11%)	9 (81.81%)	6 (60.00%)
Age, median (IQR), year	49.5035.25; 60.75)	40.00 (27.75; 50.00)	45.00 (36.00; 57.00)	37.50 (27.00; 49.75)
Disease duration, median (IQR), y	5.70 (3.28; 18.06)	9.00 (1.74; 16.19)	5.70 (4.40; 14.39)	9.45 (1.08; 15.98)
Previous biologicals, median	1 (1;1)	1 (1;1)	1 (0; 1)	1 (0;1)
Steroids at start, *n* (%)	9 (64.29%)	10 (55.56%)	7 (63.64%)	6 (60.00%)
C-reactive protein baseline, median (IQR), mg/mL	4.2 (1.0; 10.3)	1.1 (0.0; 8.6)	5.3 (2.1; 16.7)	5.5 (1.4; 17.4)
C-reactive protein endpoint, median (IQR), mg/mL	0.3 (0.3; 2.1)	5.5 (2.3; 16.5)*	0.3 (0.3; 2.05)	4.6 (1.9; 15.8)
Fecal calprotectin baseline, median (IQR), μg/g	1,355 (1,069; 1,800)	1,370 (853; 1,800)	1,355 (1,069; 1,800)	1,306 (407; 1,800)
Fecal calprotectin endpoint, median (IQR), μg/g	15 (0; 313)	56 (0; 1,800)	15 (0; 726)	120 (0; 976)
**Disease location**, ***n*** **(%)**				
Ileal (L1)	2 (33.30%)	3 (23.01%)	4 (66.67%)	3 (75.00%)
Colonic (L2)	3 (50.00%)	5 (38.46%)	1 (16.67%)	1 (25.00%)
Ileocolonic (L3)	1 (16.60%)	5 (38.46%)	1 (16.67%)	0 (0.00%)
Upper GI involvement (L4)	0 (0.00%)	0 (0.00%)	0 (0.00%)	0 (0.00%)
**Disease activity**				
B1	2 (33.30%)	3 (23.01%)	1 (20.00%)	1 (25.00%)
B2	3 (50.00%)	5 (38.46%)	2 (40.00%)	2 (50.00%)
B3	1 (16.60%)	5 (38.46%)	2 (40.00%)	1 (25.00%)
**Disease extension**, ***n*** **(%)**				
Proctitis (E1)	3 (37.50%)	1 (20.00%)	2 (50.00%)	3 (42.76%)
Left-sided (E2)	2 (25.00%)	2 (40.00%)	2 (50.00%)	2 (28.57%)
Pancolitis (E3)	3 (37.50%)	2 (40.00%)	0 (0.00%)	2 (28.57%)

Mann–Whitney U testing between responders and nonresponders; *p < 0.05, considered as significant. PBMC, peripheral blood mononuclear cell; UC, ulcerative colitis; CD, Crohn disease; GI, gastrointestinal; IQR, interquartile range.

### Altered Distribution of ILC Subsets in the Intestine and Blood of IBD Patients With Active Disease

**Figure 1:**
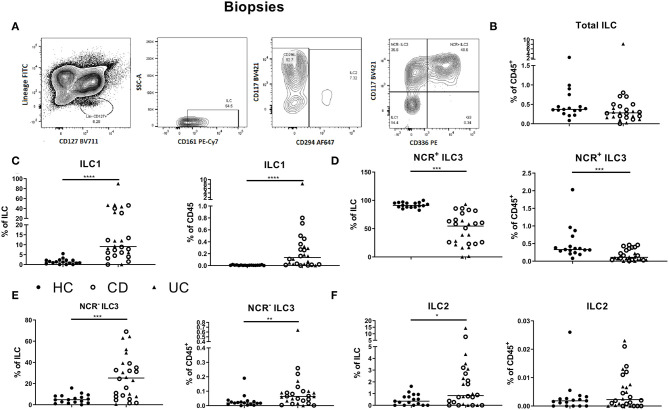
Innate lymphoid cell distribution in colonic biopsies of active IBD patients. Gating strategy for identification of innate lymphoid cells (ILCs) in intestinal biopsies. Gating was based on FMO and tonsil staining. Representative data in **(A)** are shown as contour plots (5%) with outliers **(A)**. Innate lymphoid cells were defined as living CD45^+^ lineage^−^ CD161^+^ CD127^+^ cells. Among the ILCs, ILC2 was identified by the expression of CRTH2^+^ (CD294); NCR^−^ ILC3 was defined as CRTH2^−^ CD117^+^ NKp44^−^ cells; NCR^+^ ILC3 was defined as CRTH2^−^ CD117^+^ NKp44^+^ cells and ILC1 as CRTH2^−^ CD117^−^ NKp44^−^ cells. Contribution of the ILC population to the total CD45 population isolated from the biopsies **(B)**. Comparison of ILC1 **(C)**, NCR^+^ ILC3 **(D)**, NCR^−^ ILC3 **(E)** and ILC2 **(F)** (each as proportion of total ILC and as percentage of total CD45^+^ leukocyte population) between healthy controls (*n* = 17) (left) and IBD patients (*n* = 28, CD = 15, UC = 13) (right). Mann–Whitney *U* testing; *p < 0.05, **p ≤ 0.01, ***p ≤ 0.001, and ****p < 0.0001.

To study whether active intestinal inflammation has an effect on colonic and peripheral ILC numbers and subset distribution, biopsies and blood were collected in patients with active endoscopic disease, independent of treatment and compared to gender-matched healthy controls (see demographic data of these subjects in Table 1). Innate lymphoid cells were identified by flow cytometry using a previously established gating strategy and validated with cytokine stainings (Figure 1A, Supplementary Figure 3). We confirmed that NCR^+^ ILC3 are the predominant ILC subtype in the intestinal mucosa of healthy controls (HCs) (Figure 1) (26). No differences between IBD patients and HCs were observed in total ILCs in the mucosa (0.28 vs. 0.37% of CD45, *p* = 0.076) (Figure 1B). Patients with IBD had a lower proportion of NCR^+^ ILC3 compared to HCs (54.55 vs. 91.10% of total ILCs, *p* < 0.001; 0.11 vs. 0.33% of total CD45^+^ cells, *p* < 0.001). Proinflammatory ILC1, on the other hand, were higher in IBD patients than in HCs (9.07 vs. 1.24% of total ILC; 0.28 vs. 5.5 × 10^−3^ % of total CD45, both *p* < 0.001) and the same was found for precursor NCR^−^ ILC3 (25.20 vs. 4.76% of total ILCs, *p* = 0.002; 0.06 vs. 0.02% of total CD45, *p* = 0.006; Figures 1C–F).

**Figure 2:**
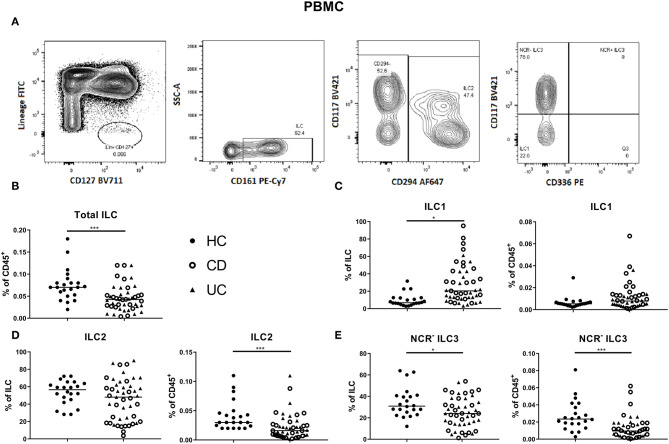
Innate lymphoid cell distribution in the blood of active IBD patients. Gating strategy of innate lymphoid cells (ILCs) among PBMC. Gating was based on FMO and tonsil staining. Representative data in **(A)** are shown as contour plots (5%) with outliers. Innate lymphoid cells were identified as described in Figure 1. Contribution of the ILC population to the total CD45 population of PBMC **(B)**. ILC1 **(C)**, ILC2 **(D)**, NCR^−^ ILC3 **(E)** [each as proportion of total ILC (left) or as percentage of total leukocyte (right) population] for healthy controls (*n* = 22) and IBD patients (*n* = 45; CD = 24 and UC = 21). Mann–Whitney *U* testing; *p < 0.05, ***p ≤ 0.001. PBMC, peripheral blood mononuclear cells; FMO, fluorescence minus one; NCR, natural cytotoxic receptor; ILC, innate lymphoid cell; CD, Crohn disease; UC, ulcerative colitis.

In circulation, ILC1, ILC2 and NCR^−^ ILC3 represent 6.81, 56.55 and 30.90% of ILCs, respectively (median values; Figures 2A–E). The proportion of total ILCs among circulating leukocytes was decreased in IBD patients (all had active disease; 0.04 vs. 0.07% of CD45^+^, *p* < 0.001; Figure 2B). Concerning subsets, we found a decrease of immature NCR^−^ ILC3 in IBD patients as compared to HCs (24.00 vs. 30.90% of total ILC population, *p* = 0.02; 0.01 vs. 0.02% of total CD45^+^ cells, *p* < 0.001), whereas the proportion of ILC1 among ILCs in blood was increased in IBD patients (20.40 vs. 6.81%, *p* < 0.0001; Figures 2C–E). Furthermore, the activation marker CD69 was expressed on 97.80% of mucosal ILCs, whereas expressed only on 2.45% of circulating ILCs, indicating that most ILCs in the circulation were in a resting state and those in tissue were activated (Supplementary Figures 4A,B). No differences were observed between UC and CD patients for any of the ILC populations neither in the intestine nor in the blood (Figures 1, 2).

### Expression of Integrin β7 and α4β7 on Circulating and Intestinal ILC

It is still debated whether ILCs in mucosal tissues are recruited from the circulation or differentiated from precursor cells *in situ* (15). To determine whether ILCs could potentially be affected by vedolizumab (anti-α4β7 integrin) therapy, we studied the expression of the β7 subunit and of α4β7 integrin on ILCs (Supplementary Figures 5A,B). In the circulation, β7 was expressed on 21.73% of total ILCs, whereas only 7.74% of all ILCs expressed α4β7 integrin. α4β7 expression was significantly higher on ILC1 as compared to ILC2 (14.55 vs. 5.10%, *p* = 0.048). ILC1 and NCR^−^ ILC3 had elevated expression of β7 as compared to ILC2 (35.34 and 23.92 vs. 3.55%, *p* = 0.028; Supplementary Figure 5C). However, in the mucosa, β7 and α4β7 were expressed on less than 5% of each ILC subtype (Supplementary Figure 5D).

### Ustekinumab Partly Restores ILC3 Levels Independent of Treatment Response

To explore whether IL-12/23 neutralization by anti-p40 (ustekinumab) treatment could impact the disturbed distribution of ILC3 and ILC1 in IBD patients, blood and biopsies from, respectively, 85 and 47 patients initiating ustekinumab treatment were prospectively collected. Demographics of these patients are summarized in Table 2. Endoscopic responders were defined as a minimal SES-CD decrease of 50%.

**Figure 3:**
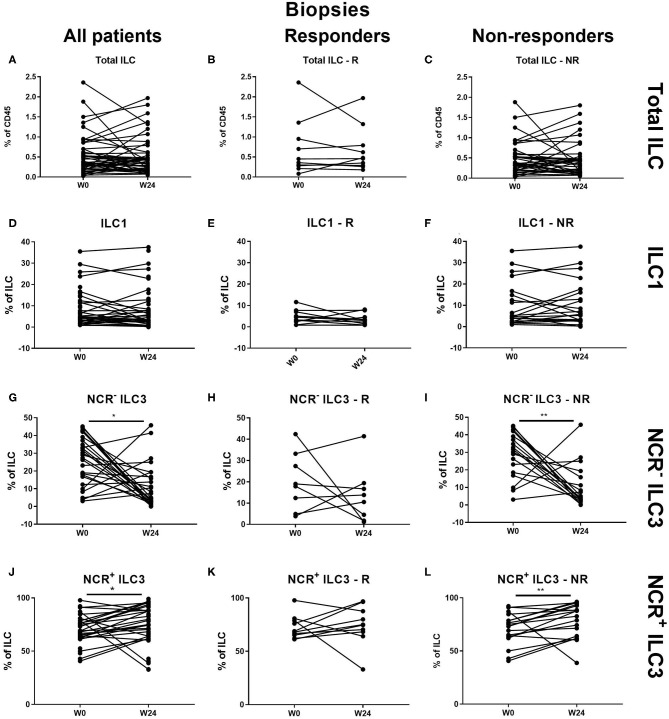
Changes in intestinal ILC subpopulations upon ustekinumab treatment. Forty-seven patients initiating ustekinumab treatment after endoscopic evaluation were subdivided into responders (R, *n* = 10) and nonresponders (NR, *n* = 37) based on endoscopic reevaluation at week 24. Paired analysis of total ILC as proportion of CD45^+^ leukocytes in all patients **(A)**, R **(B)** and NR **(C)**. Paired analysis of ILC1 as proportion of total ILC in all patients **(D)**, in R **(E)** and in NR **(F)**. Paired analysis of NCR^−^ ILC3 as proportion of total ILC in all patients **(G)**, in R **(H)** and in NR **(I)**. Paired analysis of NCR^+^ ILC3 as proportion of total ILC in all patients **(J)**, in R **(K)** and in NR **(L)**. Wilcoxon paired-match testing **(D–L)**; *p < 0.05, **p ≤ 0.01. Responders (R), nonresponders (NR) and innate lymphoid cell (ILC).

At the intestinal level, there was no effect of ustekinumab on the total ILC and ILC1 proportions over time in either responders (R) or nonresponders (NR) (Figures 3A–F). In contrast, ustekinumab treatment resulted in a slight but significant increase of the intestinal NCR^+^ ILC3 population (69.20 vs. 72.70%, *p* = 0.012). Surprisingly, this trend was not selective for therapy response (R: 68.40 vs. NR: 74.80%) and could be observed in nonresponders (73.20 vs. 78.45%, *p* = 0.006; Figures 3J–L). The increase of NCR^+^ ILC3 matched with a decrease of NCR^−^ ILC3 (27.40 vs. 13.65%, *p* = 0.032, NR: 30.20 vs. 12.26%, *p* < 0.001; Figures 3G–I).

Patients initiating ustekinumab had higher intestinal NCR^+^ ILC3 levels as compared to the reference cohort of patients with active disease (69.20 vs. 54.55% of ILCs, *p* = 0.001), but still lower when compared to healthy controls (69.20 vs. 91.10%, *p* = 0.001; Supplementary Figure 6A). No correlation could be found between intestinal ILC1 or NCR^+^ ILC3 numbers with either CRP, fCAL, or SES-CD (Supplementary Figures 6B–D).

**Figure 4:**
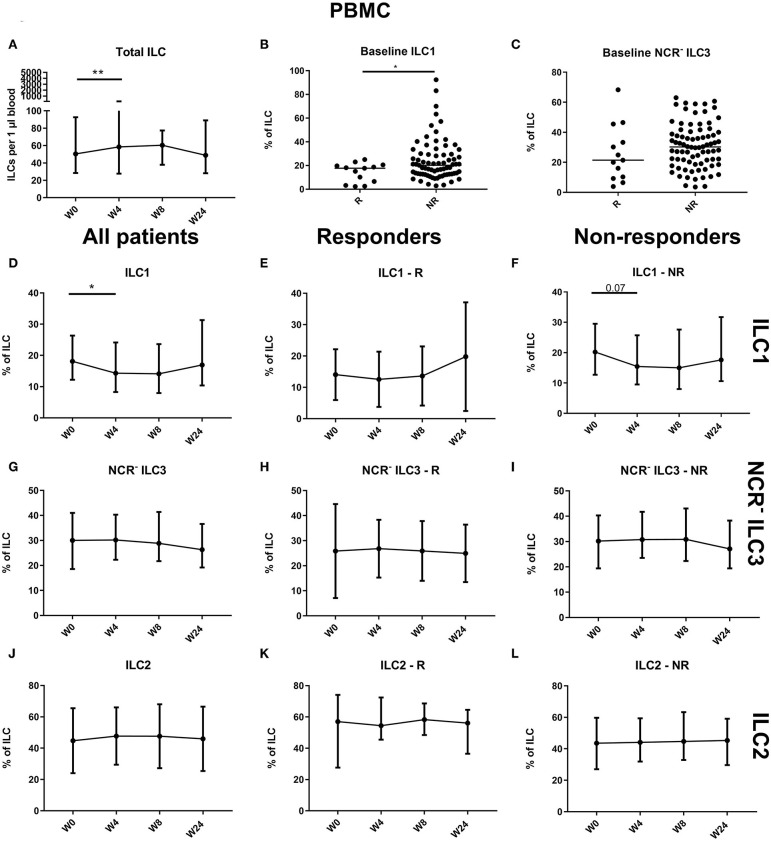
Follow-up of circulating ILC during ustekinumab treatment. Eighty-five patients initiating ustekinumab treatment were followed up for 24 weeks. Blood was collected at weeks 0, 4, 8 and 24. Patients were subdivided into responders (R, *n* = 15) and nonresponders (NR, *n* = 70) based on endoscopic evaluation. Total ILC proportion expressed as cells per microliter of blood in all patients **(A)**. Baseline ILC1 **(B)** and NCR^−^ ILC3 **(C)** levels expressed as proportion of ILC in R and NR. Follow-up of circulating ILC1 in all patients **(D)**, R **(E)** and NR **(F)**. Follow-up of circulating NCR^−^ ILC3 in all patients **(G)**, R **(H)** and NR **(I)**. Follow-up of circulating ILC2 in all patients **(J)**, R **(K)** and NR **(L)**. Mann–Whitney U testing **(B,C)**; Wilcoxon paired-match testing **(A,D–L)**; *p < 0.05, **p ≤ 0.01. NCR, natural cytotoxic receptor; ILC, innate lymphoid cell.

Comparison of blood ILC subtypes at baseline from ustekinumab-treated patients revealed increased ILC1 proportions in blood of nonresponders (NR) as compared to responders (R) (20.20 vs. 17.70%, *p* = 0.048) (Figures 4A,B). No differences between R and NR in circulating immature ILC3 (NCR^−^ ILC3) at baseline were observed (21.40 vs. 30.20%, *p* = 0.192; Figure 4C).

An initial effect of ustekinumab treatment was shown by an increased absolute ILC number at week 4 (58.50 vs. 50.38 ILCs per microliter of blood, *p* = 0.014), but this was no longer present at week 24 compared to week 0 (48.81 ILCs per microliter of blood, *p* = 0.682; Figure 4A). In nonresponders, we observed a slight increaseIn three cohorts of of the circulating ILC proportion at week 4 (0.046 vs. 0.053% of CD45, In three cohorts of *p* = 0.012), but this could no longer be seen at week 24 (0.051%, *p* = 0.1) or in the total patient population (*p* = 0.076; data not shown). Within the circulating ILC population, an initial decrease of ILC1 was observed at week 4 (18.10 vs. 14.30% of ILCs, *p* = 0.036); however, they had returned toward baseline levels at week 24 (19.95%, *p* = 0.594) (Figures 4D–F). In contrast, no changes were observed on circulating NCR^−^ ILC3 or ILC2 (Figures 4G–L).

### Anti-TNF Treatment Increases Circulating NCR^−^ILC3 Levels and Intestinal NCR^+^ILC3

To determine whether the effect on intestinal ILC3 levels was specific for ustekinumab treatment, we studied patients initiating other biological therapies, namely anti-TNF (*n* = 25) and vedolizumab (*n* = 32) treatment. Demographics of these patients are summarized in Tables 3, 4.

**Figure 5:**
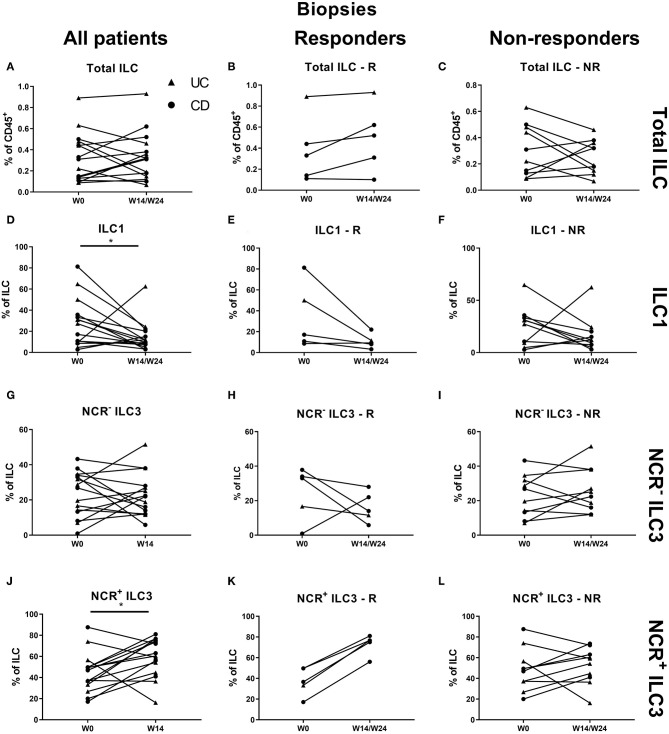
Changes in intestinal ILC and ILC subpopulations upon anti-TNF treatment. Fifteen patients initiating anti-TNF treatment after endoscopic evaluation were subdivided into responders (R, *n* = 5) and nonresponders (NR, *n* = 10) based on endoscopic reevaluation at week 14 (UC, Δ) or week 24 (CD, ∙). Paired analysis of total ILC as proportion of CD45^+^ leukocytes in all patients **(A)**, in R **(B)** and in NR **(C)**. Paired analysis of ILCI as proportion of total ILC in all patients **(D)**, in R **(E)** and in NR **(F)**. Paired analysis of NCR^−^ ILC3 as proportion of total ILC in all patients **(G)**, in R **(H)** and in NR **(I)**. Paired analysis of NCR^+^ ILC3 as proportion of total ILC in all patients **(J)**, in R **(K)** and in NR **(L)**. Wilcoxon paired-match testing **(D–L)**; *p < 0.05. NCR, natural cytotoxic receptor; ILC, innate lymphoid cell.

At the mucosal level, no change on the total ILC proportion was observed during anti-TNF treatment (Figures 5A–C). ILC1 were decreased by TNF neutralization independent of therapy response (27.30 vs. 11.60% of ILCs, *p* = 0.042), in favor of NCR^+^ ILC3 (46.90 vs. 60.40%, *p* = 0.036; Figures 5D–L).

**Figure 6:**
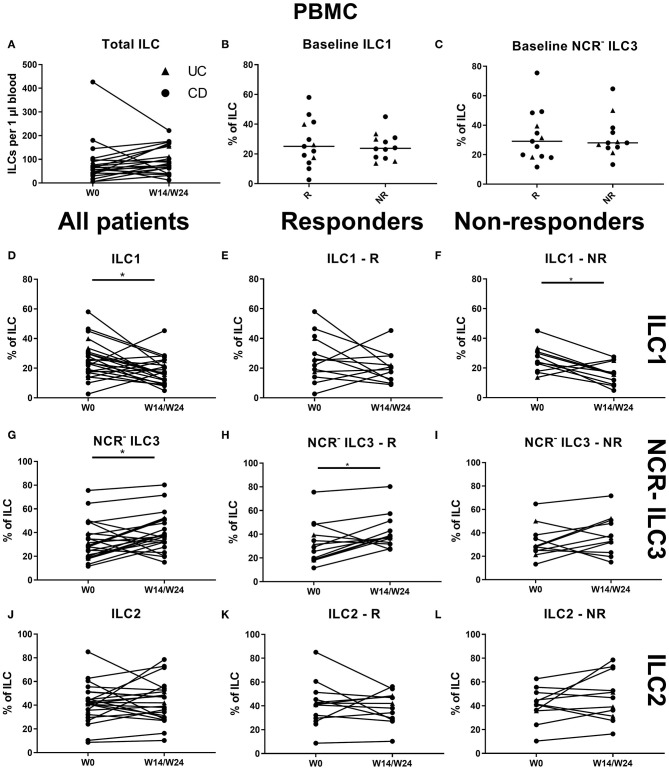
Effect of anti-TNF treatment on ILC and ILC subsets in blood. Twenty-five patients initiating anti-TNF treatment were followed up for 14 (UC, Δ)/24 (CD, ∙) weeks and subdivided into responders (R, *n* = 13) and nonresponders (NR, *n* = 12) based on endoscopic evaluation. Total ILC numbers expressed per microliter of blood in all patients **(A)**. Baseline ILC1 **(B)** and NCR^−^ ILC3 **(C)** levels in R and in NR. Follow-up of circulating ILC1 in all patients **(D)**, in R **(E)** and in NR **(F)**. Follow-up of circulating NCR^−^ ILC3 in all patients **(G)**, in R **(H)** and in NR **(I)**. Follow-up of circulating ILC2 in all patients **(J)**, in R **(K)** and in NR **(L)**. Mann–Whitney U testing **(B,C)**; Wilcoxon paired-match testing **(A,D–L)**; *p < 0.05. NCR, natural cytotoxic receptor; ILC, innate lymphoid cell CD, Crohn disease; UC, ulcerative colitis.

In contrast to the findings in the ustekinumab cohort, there was no difference in the circulating ILC1 proportion at baseline between anti-TNF nonresponders and responders (25.10 vs. 23.75% of total ILCs, *p* = 0.952) or in total ILC numbers over time (Figures 6A–C). However, TNF neutralization resulted in a decreased proportion of ILC1 in the circulation at week 14 as compared to baseline (17.06 vs. 24.10% of total ILCs, *p* = 0.023). Surprisingly, this decrease was confirmed on separate analysis of nonresponders (15.80 vs. 23.75%, *p* = 0.027) but not of responders (*p* = 0.346; Figures 6D–F). In contrast, NCR^−^ ILC3 increased upon anti-TNF treatment in responders (38.00 vs. 29.10% of total ILCs, *p* = 0.032), whereas such an effect was not observed in nonresponders (*p* = 0.272; Figures 6G–I). No differences were observed in ILC2 (Figures 6J–L).

### Vedolizumab Therapy Increases Circulating ILC3 and Intestinal NCR^+^ ILC3

**Figure 7:**
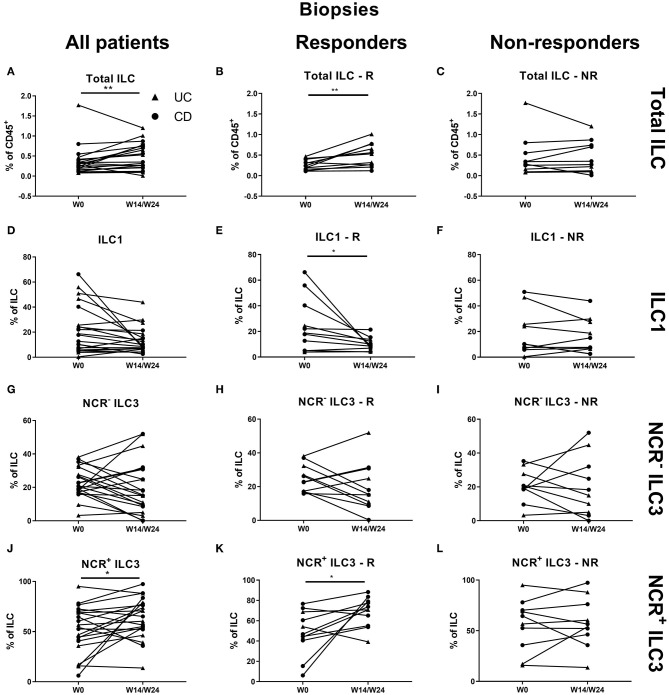
Effect of vedolizumab therapy on intestinal ILC and ILC subsets. Twenty-one patients initiating vedolizumab treatment were followed up for 14 (UC, Δ)/24 (CD, ∙) weeks and subdivided into responders (R, *n* = 11) and nonresponders (NR, *n* = 10) based on endoscopic evaluation. Paired analysis of total ILC as proportion of CD45^+^ leukocytes in all patients **(A)**, R **(B)** and NR **(C)**. Paired analysis of ILCI as proportion of total ILC in all patients **(D)**, in R **(E)** and in NR **(F)**. Paired analysis of NCR^−^ ILC3 as proportion of total ILC in all patients **(G)**, in R **(H)** and in NR **(I)**. Paired analysis of NCR^+^ ILC3 as proportion of total ILC in all patients **(J)**, in R **(K)** and in NR **(L)**. Wilcoxon paired-match testing **(D–L)**; *p < 0.05, **p ≤ 0.01. NCR, natural cytotoxic receptor; ILC, innate lymphoid cell; CD, Crohn disease; UC, ulcerative colitis.

Treatment with vedolizumab resulted in an increased proportion of ILCs in intestinal biopsies (0.28 vs. 0.35% of CD45, *p* = 0.009, R: 0.24 vs. 0.53%, *p* = 0.005; Figures 7A–C). Within this increased ILC proportion, a decrease of ILC1 (17.80 vs. 9.00% of ILCs, *p* = 0.018) and an increase of NCR^+^ ILC3 (54.20 vs. 65.10% of ILCs, *p* = 0.049) was observed. This shift was specific for responders (ILC1: 18.78 vs. 9%, *p* = 0.012; NCR^+^ ILC3: 46.80 vs. 70.80%, *p* = 0.021; Figures 7D–L).

**Figure 8:**
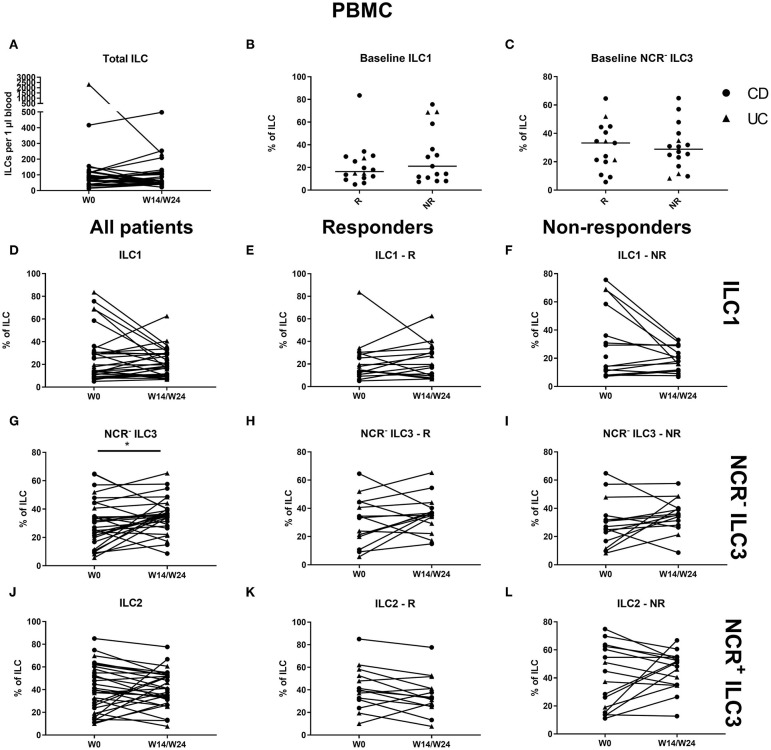
Changes in circulating ILC and ILC subpopulations during vedolizumab therapy. Thirty-two patients initiating vedolizumab treatment were followed up for 14 (UC, Δ)/24 (CD, ∙) weeks and subdivided into responders (R, *n* = 14) and nonresponders (NR, *n* = 18) based on endoscopic evaluation. Total ILC expressed as cells per microliter of full blood in all patients **(A)**. Baseline ILC1 **(B)** and NCR^−^ ILC3 **(C)** levels in R and in NR. Follow-up of circulating ILC1 in all patients **(D)**, in R **(E)** and in NR **(F)**. Follow-up of circulating NCR^−^ ILC3 in all patients **(G)**, R **(H)** and NR **(I)**. Follow-up of circulating ILC2 in all patients **(J)**, in R **(K)** and in NR **(L)**. Mann–Whitney *U* testing **(B,C)**; Wilcoxon paired-match testing **(A,D–L)**; *p < 0.05. NCR, natural cytotoxic receptor; ILC, innate lymphoid cell; CD, Crohn disease; UC, ulcerative colitis.

No effect of vedolizumab was seen on the total ILC pool in the circulation and no baseline ILC1 differences were observed between responders and nonresponders (Figures 8A–C). Circulating ILC1 and ILC2 also did not change, whereas NCR^−^ ILC3 was increased at week 14 (36.59 vs. 30.6% of total ILCs, *p* = 0.035; Figures 8D–L).

### Increase of Peripheral NCR^+^ ILC3 Levels During Biological Treatment

**Figure 9:**
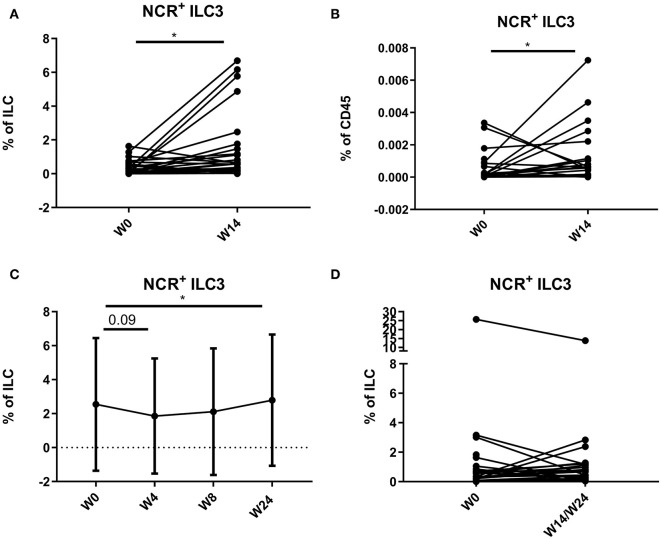
Mobilization of NCR^+^ ILC3 in the circulation of patients treated with anti-TNF, ustekinumab, or vedolizumab. Paired analysis of NCR^+^ ILC3 as proportion of total ILC **(A)** and of total leukocytes **(B)** in anti-TNF–treated patients before and after treatment. Follow-up of NCR^+^ ILC3 as proportion of total ILC in ustekinumab **(C)**–treated patients. Paired analysis of NCR^+^ ILC3 as proportion of total ILC in vedolizumab **(D)**–treated patients before and after treatment. Wilcoxon paired-match testing; *p < 0.05. NCR, natural cytotoxic receptor; ILC, innate lymphoid cell.

In 18 CD patients (15.38%), more than 3% of the total ILC proportion in the circulation consisted of NCR^+^ ILC3, a population not previously detected in blood in healthy individuals or IBD patients. In contrast, in none of the UC patients (0/23) circulating NCR^+^ ILC3s were detected. A further increase of peripheral NCR^+^ ILC3 among the total ILCs and among the total leukocyte population could be observed in the anti-TNF–treated subjects (respectively, 0.18 vs. 0.62%, *p* = 0.015; and 1.00 vs. 5.73 per 10,000 CD45^+^ cells, *p* = 0.038) and to a lesser extent in ustekinumab-treated subjects (0.93 vs. 1.18 % of ILCs, *p* = 0.042) as compared to the start of therapy (Figures 9A–C). In contrast, no effect of vedolizumab treatment (*p* = 0.474) was observed on peripheral NCR^+^ ILC3 levels (Figure 9D).

## Discussion

In this study, we analyzed the distribution of ILC subsets in intestinal tissues and blood of IBD patients and compared this to the distribution in healthy controls. We could reproduce the results from Bernink et al. (14) and Forkel et al. (27), who showed that the dominant ILC population in the healthy gut mucosa is the NCR^+^ (NKp44^+^) ILC3 subset. In intestinal lesions of IBD patients, we confirmed that there is a subset shift with a lower proportion of NKp44^+^ ILC3, on the one hand and more ILC1 and more NCR^−^ (NKp44^−^) ILC3, on the other hand (14, 27). Despite these subset shifts, we have no evidence for expansion of the total ILC population in IBD, suggesting that the altered ratio between subsets results from a change in the differentiation and/or recruitment of particular subsets. These results were similar in UC and CD patients, despite the current thinking that both variants of IBD have a different immunologic background (4).

Up until now, the effect of treatment on ILCs in IBD patients has hardly been studied (27). In three cohorts of ustekinumab-, anti-TNF–, or vedolizumab-treated patients, we found a partial restoration toward normal of the altered mucosal ILC subset ratio with an increase of NKp44^+^ ILC3 (with all three biologicals) and a decrease of ILC1 (with anti-TNF and vedolizumab). Moreover, this shift in vedolizumab-treated patients was specific for treatment responders and coincided with a significantly decreased ILC1 and increased NKp44^+^ ILC3 proportion. A relative increase of the total intestinal ILC pool among CD45^+^ leukocytes was also observed in vedolizumab-treated patients. The changes observed in ustekinumab and anti-TNF–treated patients were significant in the total group and in the nonresponder group, but not when the responder population was analyzed separately. The nonsignificance of the results in responders is surprising but might relate to the small sample size resulting in limited power. As our center is a tertiary health care institute, ustekinumab-treated patients were mainly refractory patients with a history of failure on previous biologicals. The limited response to ustekinumab is therefore not surprising but differs from results in other studies (21, 28). Validation of our results in newly diagnosed/biological–naive patients is required.

We also had the opportunity to analyze ILC subsets in blood of a large group of IBD patients and we found again IBD disease–associated changes when compared with HCs. No change in absolute numbers of ILCs was found, but compared to controls, IBD patients had an elevated proportion of circulating ILC1 (similar to what is found in the intestine), whereas circulating NKp44^−^ ILC3 were decreased. The latter probably reflects a reduction of circulating ILC precursors. Indeed, Lim et al. (16) could show that, after *in vitro* stimulation, these circulating NKp44^−^ ILC3 can differentiate toward all subtypes of ILCs depending on the cytokine stimulation mix. The change in circulating ILC1 and NKp44^−^ ILC3 is somehow similar to what is found in the gut mucosa, but there is also an important difference, as almost no NKp44^+^ ILC3 can be found in circulation, although this is the predominant population in the mucosa. Importantly, we are the first to report that CD patients are an exception to this. We detected NKp44^+^ ILC3 in the circulation of 15% of CD patients, although they are absent in all UC patients and HCs. Presence of these NKp44^+^ ILC3 cells in the circulation did not correlate with disease severity or extraintestinal symptoms. Strikingly, treatment with anti-TNF (and to a lesser extent) ustekinumab resulted in a further increase of NKp44^+^ ILC3 in the circulation over time. As CD is a transmural inflammation, we can speculate that there is a spillover of mucosal ILCs into the circulation as it has also been observed for CD4^+^ and CD8^+^ T cells (29). Another possibility is that these cells are generated in other lymphoid organs, released in circulation and then recruited to the mucosa to assist in healing. Other possibilities to explain the rise of this subset in the blood during treatment should be explored.

During biological treatment, there were also some other changes in circulating ILC subsets. There was an increase of the circulating NKp44^−^ ILC3 population in anti-TNF and vedolizumab-treated patients and a decrease of the ILC1 proportion in anti-TNF–treated patient. These results are in contrast with observations made by Forkel et al. (27); however, a trend toward higher NKp44^−^ ILC3 is also visible in their study and discrepancies might be explained by a shorter period of follow-up after vedolizumab initiation in their study. A small decrease of circulating ILC1 in ustekinumab-treated patients was transient and limited to the first 4 weeks of treatment. The increase of NKp44^−^ ILC3 was absent in ustekinumab-treated patients, again correlating with the poor clinical efficacy of ustekinumab in this cohort. As circulating NKp44^−^ ILC3 can differentiate toward all subtypes of ILCs, the increase of NKp44^−^ ILC3 cells can be seen as a restoration of the ILC precursor pool.

The observed effects of biologicals on intestinal ILCs (decrease of ILC1 and increase of NKp44^+^ ILC3) might contribute to their clinical efficacy. It remains, however, to be explored why these changes in ILCs occurred. The results were unexpected for the anti-TNF and vedolizumab treatment, as we had originally included these groups as reference groups. Our underlying hypothesis was that only ustekinumab would have an effect (based on its p40 neutralizing effect). Interleukin 12 has been shown *in vitro* and *in vivo* to be essential for ILC1 differentiation and expansion (14, 30, 31). Differentiation toward ILC3 is observed when ILC1 or ILC precursors are cultured in presence of IL-23 and IL-1β (14). As ustekinumab elicits its beneficial effect by neutralization of both IL-12 and IL-23, we had expected a decrease of both ILC1 and mature ILC3. In fact, none of these occurred. The effect of p19-directed IL-23-specific antibodies such as risankizumab, brazikumab, guselkumab and mirikizumab, currently in phases II and III trials in CD and UC, on the intestinal ILC1/ILC3 ratio might make it more clear whether IL-23 affects mature ILCs *in vivo* (32, 33). It then remains to be explored how the other biologicals act on ILCs. Several possibilities can be considered and await further experimental approaches. Tumor necrosis factor, a key mediator of intestinal inflammation in IBD, can be produced by ILC1 (34). As TNF is secreted in a membrane form, anti-TNF treatment may directly impact the function of ILC1 (35, 36) similar to its direct impact on macrophages (37). The strong effect of vedolizumab on ILC1 could be explained by reduced recruitment from circulation, but this provides no explanation for the observed rise in NKp44^+^ ILC3. In fact, the results with vedolizumab suggest an alternative explanation. We favor the possibility that the changes in ILC subsets result from reduced inflammation and not from a direct effect of the biologicals on ILCs. As we saw the best therapeutic effect with vedolizumab and anti-TNF (approximately 50% responders) and a weak therapeutic effect with ustekinumab (13% responders), this therapeutic effect in fact correlates with the extent of changes in ILC subsets (which were most clear with vedolizumab and least with ustekinumab). We therefore think that it is unlikely that the changes in ILC subsets directly reflect the mechanism of action of the biologicals. We more likely observed a collateral effect on the ILC subset distribution due to intestinal healing during therapy.

In summary, we found that different biological treatments can partly restore the disturbed intestinal ILC subset levels, independent of the mode of action, whereas ILC subset changes in the circulation were limited. Importantly, we report the presence of NKp44^+^ ILC3 in the circulation of a subset CD (but not UC) patients. As ILC1 is proinflammatory cells and as NKp44^+^ ILC3 contribute to homeostasis of intestinal mucosa, the observed effects on intestinal ILCs during treatment might contribute to the clinical efficacy of these biologicals.

## Data Availability Statement

All datasets generated for this study are included in the article/Supplementary Material.

## Ethics Statement

Blood and tissue samples were collected using a prospective study protocol approved by the UZ Leuven Ethical Committee Review Board (S53684). Informed consent was obtained from healthy controls (who were included upon negative endoscopic findings during polyp screening) and from adult IBD patients with confirmed diagnosis of inflammatory bowel disease.

## Author Contributions

All authors made substantial contributions to the submitted work. BC contributed to the study concept and design, acquisition of data, analysis and interpretation of data, drafting of the manuscript, and statistical analysis. IJ acquisition of data, analysis and interpretation of data. BV study concept and design, acquisition of data, analysis and interpretation of data, drafting of the manuscript, and statistical analysis. JC acquisition of data, analysis and interpretation of data. RV acquisition of data, analysis and interpretation of data. TV acquisition of data, analysis and interpretation of data, and critical revision of the manuscript for important intellectual content. MF and SV acquisition of the data, interpretation of data, and critical revision of the manuscript for important intellectual content. JLC study concept and design, interpretation of data, and critical revision of the manuscript for important intellectual content. GV study concept and design, interpretation of data, and critical revision of the manuscript for important intellectual content. CB study concept and design, analysis and interpretation of data, material support, drafting of the manuscript, and study supervision. All authors agreed with the final version of the manuscript. CB guarantor of the manuscript.

## Conflict of Interest

BV reports financial support for research from Pfizer; lecture fees from Abbvie, Ferring, Takeda Pharmaceuticals, Janssen and R Biopharm; consultancy fees from Janssen and Sandoz. TV reports consultancy fees from Takeda, Shire, Boehringer-Ingelheim, VectivBio and Falk. Lecture fees from Abbott. MF received financial support for research from Amgen, Biogen, Pfizer, Takeda and Janssen; lecture fees from Abbvie, Amgen, Biogen, Boehringer-Ingelheim, Falk, Ferring, Janssen, Lamepro, MSD, Mylan, Pfizer, Takeda; consultancy fees from Abbvie, Boehringer-Ingelheim, Janssen, MSD, Pfizer, Sandoz and Takeda. SV reports financial support for research from MSD, AbbVie, Takeda, Pfizer, J&J; lecture fees from MSD, AbbVie, Takeda, Ferring, Centocor, Hospira, Pfizer, J&J, Genentech/Roche; consultancy fees from: MSD, AbbVie, Takeda, Ferring, Centocor, Hospira, Pfizer, J&J, Genentech/Roche, Celgene, Mundipharma, Celltrion, Second Genome, Prometheus, Shire, Prodigest, Gilead, Galapagos. GV reports financial support for research from Abbott and Ferring Pharmaceuticals; lecture fees from Janssen, MSD and Abbott; consultancy fees from PDL BioPharma, UCB Pharma, Sanofi-Aventis, Abbott, Abbvie, Ferring, Novartis, Biogen Idec, Janssen Biologics, NovoNordisk, Zealand Pharma A/S, Millenium/Takeda, Shire, Novartis and Bristol Mayer Squibb. CB reports consultancy fees from Ablynx. The remaining authors declare that the research was conducted in the absence of any commercial or financial relationships that could be construed as a potential conflict of interest.
