# Supplementary material for: Biological Therapy in Inflammatory Bowel Disease Patients Partly Restores Intestinal Innate Lymphoid Cell Subtype Equilibrium
Source: Front Immunol. 2020 Aug 27;11:1847. doi: 10.3389/fimmu.2020.01847 (PMC7481382; doi:10.3389/fimmu.2020.01847)
Supplement: Supplementary file 1 [file Data_Sheet_1.docx]

Biological Therapy in Inflammatory Bowel Disease Patients Partly Restores Intestinal Innate Lymphoid Cell Subtype Equilibrium - Supplementary Material

**Supplementary figure 1:**

**Supplementary figure 1.** ILC subtype distribution before and after cryopreservation of PBMC.

PBMCs were stained for ILC subtyping freshly after isolation and after 24 weeks of cryopreservation. Data of ILC subtypes are presented as percentage of living CD45^+^ Lineage^-^ CD161^+^ CD127^+^ ILC. Wilcoxon paired testing, n = 10; (* p<0.05, ** p≤0.01, *** p≤0.001). Peripheral blood mononuclear cell (PBMC); Innate lymphoid cell (ILC).

**Supplementary figure 2:**

 **Supplementary figure 2.** Characteristics of recruited patients initiating different biological therapies

Age (A), disease duration (B) and previous biological treatment exposure (C) at start of treatment. Data are expressed as individual values with medians. Kruskal-Wallis testing is shown over all groups. Multiple comparison with ustekinumab is shown above the individual group. (* p<0.05, ** p≤0.01, *** p≤0.001). Ustekinumab (UST); Vedolizumab (VDZ); Tumor Necrosis Factor (TNF).

**Supplementary figure 3:**

**
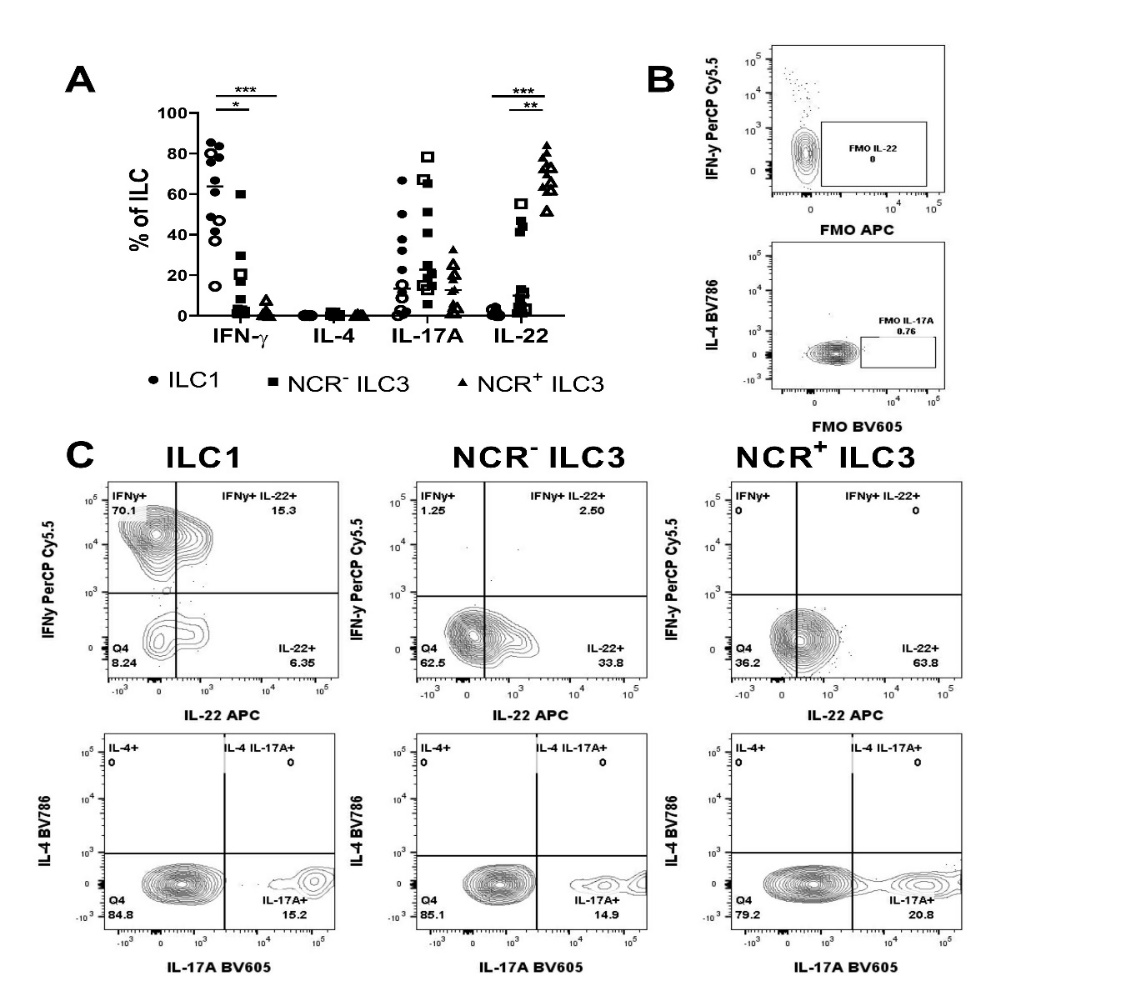
**

**Supplementary figure 3.** Expression of cytokines by intestinal ILC subsets

Mucosal biopsies from resection specimen were taken from 4 IBD, both inflamed (open symbols) and uninflamed mucosa (closed symbols) and 4 control patients. Lamina propria cells were isolated and stimulated for 6 hours with PMA/ionomycin and then stained for intracellular IFN-ɣ, IL-4, IL-17A and IL-22 and for membrane markers to identify ILC subsets. (A) shows the percentage of ILC1, NCR^-^ ILC3 and NCR^+^ ILC3 expressing IFN-ɣ, IL-4, IL-17A or IL-22 in inflamed mucosal biopsies (n=4, open symbol) and in uninflamed mucosal biopsies (n=8, closed symbol). (B) shows FMO control for IL-22 staining (top) and for IL-17A staining (bottom) on gated total ILC, identified as described in Figure 1. (C) shows representative data of staining for IFN-ɣ, IL-4, IL-17A or IL-22 for ILC1 (left), NCR^-^ ILC3 (middle) and NCR^+^ ILC3 (right) (C). Representative data in B and C are shown as contour plots (5%) with outliers. Data in (A) are shown as median with interquartile range. Kruskal-Wallis test with Dunn's multiple comparisons test is shown above the group. (* p<0.05, ** p≤0.01, *** p≤0.001). Inflammatory bowel disease (IBD), Innate lymphoid cell (ILC); Interferon (IFN), Interleukin (IL); Natural cytotoxic receptor (NCR).

**Supplementary figure 4:**

**Supplementary figure 4.** Expression of activation marker CD69 on peripheral and intestinal ILC

Mucosal biopsies and PBMC were taken from 5 IBD patients with active inflammation and stained for CD69 expression on ILC. Representative contour plots (5%) with outliers are shown in (A). Percentage of CD4^+^ cells, ILC and ILC subsets expressing CD69 among PBMC (n=4) and in mucosal biopsies (n=5) (B). Data in (B) are shown as individual values. Fluorescence minus one (FMO); Peripheral blood mononuclear cells (PBMC); Innate lymphoid cell (ILC); Inflammatory bowel disease (IBD).

**Supplementary figure 5:**

**Supplementary Figure 5.** Expression of gut homing integrin β7 and α4β7 on peripheral and intestinal ILC.

Mucosal biopsies (n=5) and PBMC (n=4) were taken from IBD patients with active inflammation and stained for β7 and α4β7 expression on ILC. Representative contour plots (5%) with outliers showing expression of β7 (A) and α4β7 (B) on mucosal and peripheral blood ILC. Percentage of CD4^+^ cells, ILC and ILC subsets expressing β7 and α4β7 among PBMC (C) and in mucosal biopsies (D). In C and D, data is shown as individual values (C,D). Fluorescence minus one (FMO); Peripheral blood mononuclear cells (PBMC); Innate lymphoid cell (ILC); Inflammatory bowel disease (IBD).

**Supplementary figure 6:**

**Supplementary figure 6.** Baseline intestinal ILC levels and their correlation with clinical markers.

Intestinal ILC in ustekinumab initiating patients (n=47) were compared to intestinal ILC in patients in a separate cohort with active disease (n=26) and in healthy controls (HC) (n=17). Correlation of ILC1 and NCR^+^ ILC3 levels with C-reactive protein (CRP) (A), faecal calprotectin (fCAL) (B) and Simple Endoscopic Score CD (SES-CD) (C). (A) Multiple comparison. (B, C, D) Spearman correlation. Healthy controls (HC), Ustekinumab (UST).
